# Transcriptional Analysis of Nuclear-Encoded Mitochondrial Genes in Eight Neurodegenerative Disorders: The Analysis of Seven Diseases in Reference to Friedreich’s Ataxia

**DOI:** 10.3389/fgene.2021.749792

**Published:** 2021-12-20

**Authors:** Muhammad Elsadany, Reem A. Elghaish, Aya S. Khalil, Alaa S. Ahmed, Rana H. Mansour, Eman Badr, Menattallah Elserafy

**Affiliations:** ^1^ University of Science and Technology, Zewail City of Science and Technology, Giza, Egypt; ^2^ Center for Genomics, Helmy Institute for Medical Sciences, Zewail City of Science and Technology, Giza, Egypt; ^3^ Faculty of Computers and Artificial Intelligence, Cairo University, Giza, Egypt

**Keywords:** friedreich ataxia (FRDA), amytrophic lateral sclerosis (ALS), alzheimers disease (AD), creutzfeld-jakob disease, frontotemporal dementia (FTD), huntington’s disease (HD), multiple sclerosis, Parkinson’s disease (PD)

## Abstract

Neurodegenerative diseases (NDDs) are challenging to understand, diagnose, and treat. Revealing the genomic and transcriptomic changes in NDDs contributes greatly to the understanding of the diseases, their causes, and development. Moreover, it enables more precise genetic diagnosis and novel drug target identification that could potentially treat the diseases or at least ease the symptoms. In this study, we analyzed the transcriptional changes of nuclear-encoded mitochondrial (NEM) genes in eight NDDs to specifically address the association of these genes with the diseases. Previous studies show strong links between defects in NEM genes and neurodegeneration, yet connecting specific genes with NDDs is not well studied. Friedreich’s ataxia (FRDA) is an NDD that cannot be treated effectively; therefore, we focused first on FRDA and compared the outcome with seven other NDDs, including Alzheimer’s disease, amyotrophic lateral sclerosis, Creutzfeldt–Jakob disease, frontotemporal dementia, Huntington’s disease, multiple sclerosis, and Parkinson’s disease. First, weighted correlation network analysis was performed on an FRDA RNA-Seq data set, focusing only on NEM genes. We then carried out differential gene expression analysis and pathway enrichment analysis to pinpoint differentially expressed genes that are potentially associated with one or more of the analyzed NDDs. Our findings propose a strong link between NEM genes and NDDs and suggest that our identified candidate genes can be potentially used as diagnostic markers and therapeutic targets.

## 1 Introduction

Neurodegenerative diseases (NDDs) are multifactorial disorders that are known to progress gradually with perceptible loss of neuronal function (Angelini et al., 2009). Although each disease has its own molecular pathological mechanism and clinical manifestations, they share a range of pathways that are thought to overlap and contribute to neuronal death (Hussain et al., 2018). The complexity of the NDDs makes it very challenging to pinpoint specific defects associated with the diseases’ development. Therefore, a lot of research efforts are still needed to reveal the genetic determinants of NDDs to improve their diagnosis and treatment. Even though recent studies conclude that mitochondrial dysfunction is a common overlapping feature between all neurodegenerative diseases, clear links between genes playing a role in mitochondrial function and NDDs are not well established (Huang et al., 2020).

The human mitochondrial proteome includes more than 1500 proteins, only 13 of which are mitochondrially encoded. The remaining mitochondrial proteins are nuclear encoded (Neupert, 2015; Wiedemann and Pfanner, 2017). In this study, we focused on Friedreich’s ataxia (FRDA), which is an autosomal recessive disorder and the most common inherited ataxia (Hart and Schapira, 2013). We compared the transcriptomic changes in nuclear-encoded mitochondrial (NEM) genes between FRDA and other seven NDDs in an attempt to identify common dysregulated genes and connect what is known about the diseases to FRDA. The diseases analyzed included Alzheimer’s disease (AD), amyotrophic lateral sclerosis (ALS), Creutzfeldt–Jakob disease (CJD), frontotemporal dementia (FTD), Huntington’s disease (HD), multiple sclerosis (MS), and Parkinson’s disease (PD). We discuss below some known links between the eight NDDs and mitochondrial dysfunction.

FRDA is triggered by an inadequate amount of an NEM protein known as frataxin, which has a vital role in iron metabolism and proper mitochondrial function (Llorens et al., 2019). About 90% of FRDA patients have an unstable GAA trinucleotide repeat in the first intron of both alleles of the FXN gene that encodes the frataxin protein (Campuzano et al., 1996). FRDA is a disease that cannot be treated effectively, which encouraged us to analyze the transcriptomic changes in NEM genes in FRDA patients and also other NDDs as a step towards identifying common genetic associations and potential drug targets.

We were keen to include diseases that are previously reported to be associated with mitochondria dysfunction. The dysfunction of mitochondria is considered to be an early feature of AD, as the Aβ protein is imported into the mitochondria via the TOM machinery (Picone et al., 2014). Aβ-induced mitochondrial dysfunction is associated with neuronal damage and cognitive decline (Swerdlow et al., 2010). In addition, it causes defects in key respiratory enzymes, accumulation of mitochondrial reactive oxygen species (ROS), and altered mitochondrial biogenesis (Moreira et al., 2010). As for ALS, even though the most common genetic cause of the disease is the expansion of the hexanucleotide intronic repeat GGGGCC in the *C9orf72* gene (Ghasemi and Brown, 2018)*,* mutations in the NEM gene SOD1 are considered to be the second most frequent cause of ALS (Bernard et al., 2020). Disruption of the mitochondrial structure and network are reported in the vast majority of ALS patients by multiple studies (reviewed in Smith et al., 2019). Moreover, these mitochondrial structural and functional impairments are reported in early ALS stages, suggesting that mitochondrial dysfunction could be an upstream cause of ALS degeneration rather than a consequence (Wang et al., 2013; Magrané et al., 2014).

Regarding FTD, the neurons are reported to exhibit a higher rate of mitochondrial ROS production and lipid peroxidation compared to controls, which leads to exacerbated damage due to oxidative stress. This ultimately leads to neuronal death by either apoptosis or necrosis (Bourens et al., 2013). Multiple studies note abnormalities in structure and function of mitochondria in FTD patients, but the exact mechanisms of mitochondrial dysfunction in FTD are yet to be elucidated.

In HD, several studies provide evidence for a link between the mutant Huntingtin (mHTT) protein and mitochondrial abnormalities that could ultimately lead to neuronal damage and degeneration in affected brain regions (Li et al., 2020). Mitochondrial dysfunction in HD may occur through aberrant transcriptional regulation as mHTT binds to several transcriptional regulators and interferes with their function (Dong and Cong, 2018). In particular, expression of PGC-1α, a master transcriptional co-regulator of mitochondrial biogenesis and antioxidant enzymes, is reduced in HD, which may contribute to mitochondrial impairment (Scarpulla, 2011; Johri et al., 2013).

Although the exact etiology of MS is still unknown, previous studies report increasing evidence that mitochondrial dysfunction is a common underlying theme of MS; ultimately leading to axonal degeneration (reviewed in Su et al., 2009). This mitochondrial dysfunction is manifested in dysregulation of Ca^2+^ homeostasis, ROS levels, and opening of the permeability transition pore. The characteristic chronic neuro-inflammatory stimuli in MS impair the neuro-axonal homeostasis and cause increased ROS-caused oxidative stress, which further damages the mitochondria and initiates a vicious cycle (Barcelos et al., 2019). In CJD, mitochondrial degeneration and electron-dense bodies of lysosomes within degenerating axons are observed (Liberski et al., 2005, 2010). Recent proteomics and gene expression studies suggest an altered gene expression pattern in proteins linked to oxidative phosphorylation (Ansoleaga et al., 2016). Mitochondrial dysfunction is also linked to PD diagnosis and progression (Gaweda-Walerych and Zekanowski, 2014).

In this study, we report transcriptional changes in multiple NEM genes for the eight NDDs of interest and highlight the common dysregulated genes and pathways with a focus on FRDA.

## 2 Results

**FIGURE 1:**
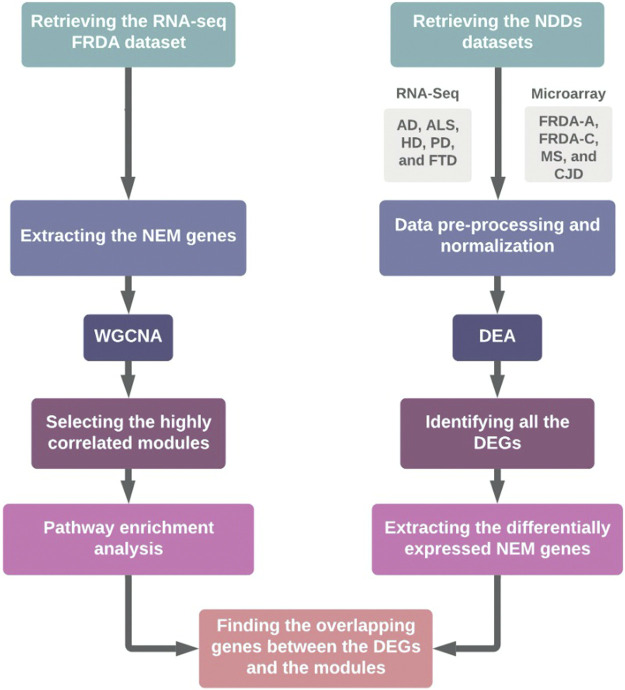
Schematic representation of the workflow. FRDA-A, FRDA-adults; FRDA-C, FRDA-children; DEA, differential expression analysis.

In this study, we aimed at linking transcriptional changes in NEM genes to FRDA and seven other NDDs. This was done through weighted gene co-expression network analysis (WGCNA) and differential expression analysis (DEA) of microarray and RNA-sequencing (RNA-seq). A detailed description of the workflow is represented in Figure 1. The data sets used for each disease and the number of patients and control samples are indicated in Table 1.

**TABLE 1:** The data sets analyzed in the study. The GEO accession ID and the number of patient and control samples are indicated.

disease	GEO accession ID	Patients’ samples	Control samples	Total samples
AD	GSE153873^1^	12	18 (10 old, 8 young)	30
HD	GSE64810^2^	20	49	69
PD	GSE68719^3^	29	44	73
FRDA (RNA-Seq)	GSE104288^4^	18	17	35
FRDA (microarray)	GSE11204^5^	42 (14 adults, 28 children)	25 (15 adults, 10 children)	67
MS	GSE135511^6^	40	10	50
CJD	GSE124571^7^	10	10	20
ALS	GSE124439^8^	148	17	165
FTD/FTLD-TDP	GSE153960^9^	63	59	122

1 http://www.funrich.org/

2 https://bioconductor.org/packages/release/bioc/html/ReactomeContentService4R.html

3 http://bioinformatics.psb.ugent.be/webtools/Venn/

4 http://www.biomart.org/

5 https://useast.ensembl.org/index.html

6 https://www.genecards.org/

7 https://bioconductor.org/packages/release/data/annotation/html/org.Hs.eg.db.html

8 https://www.ncbi.nlm.nih.gov/geo/

9 https://cran.r-project.org/web/packages/WGCNA/index.html

10 https://www.molbiotools.com

First, WGCNA was employed to identify the gene co-expression modules in FRDA. The analysis was performed on RNA-seq data of FRDA patients and normal samples (GSE104288) (Napierala et al., 2017). We analyzed only the NEM genes to link dysregulation of mitochondrial dysfunction to the disease. Around 1650 NEM genes retrieved from Eslamieh et al. (2017) were analyzed (full list provided in Supplementary Table S1). We also checked the confidence (conf.) score for mitochondrial localization for all proteins encoded by the genes (Supplementary Table S1) (Binder et al., 2014).

**FIGURE 2:**
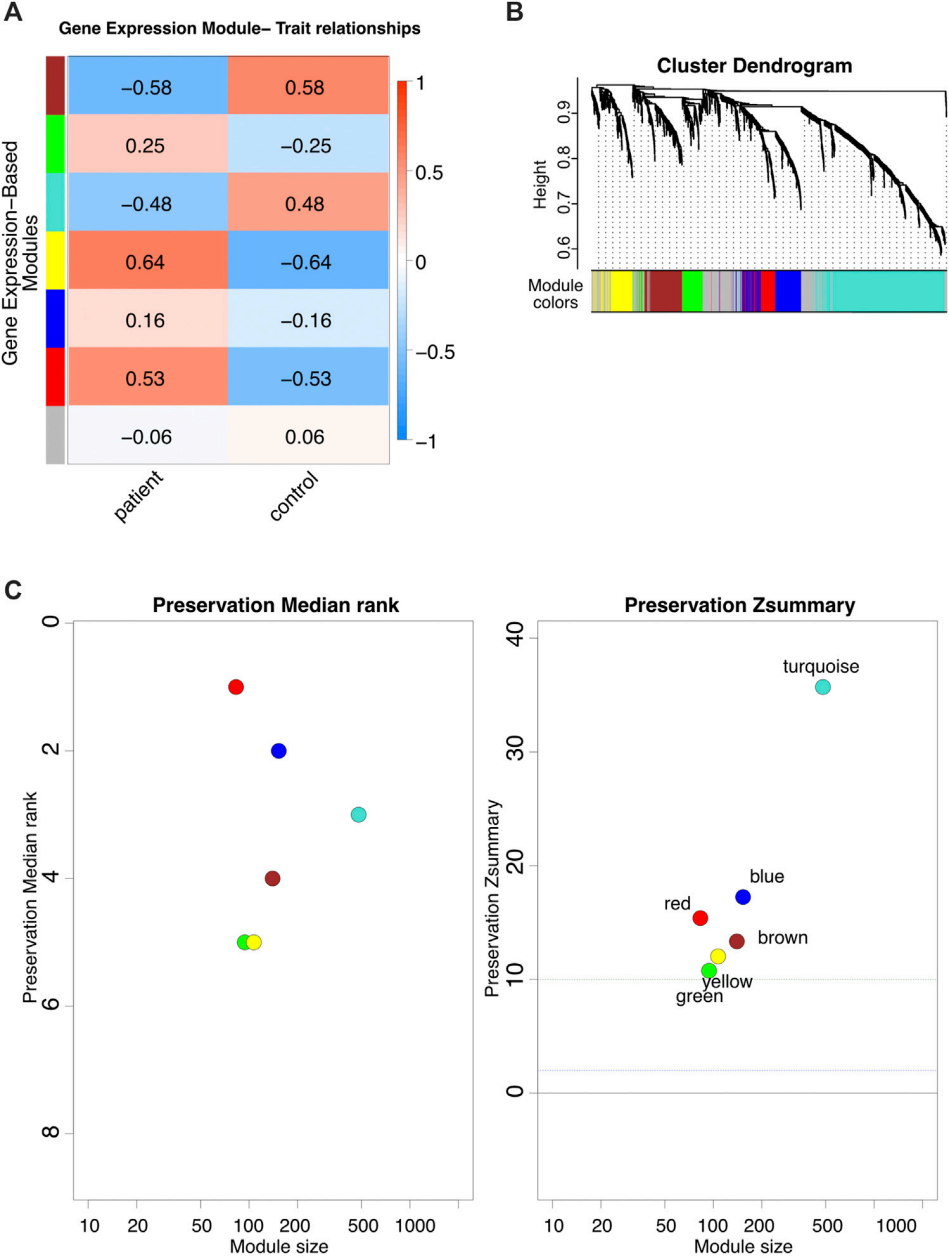
WGCNA analysis. **(A)** The modules identified via WGCNA on FRDA RNA-seq dataset. **(B)** Dynamic dendrogram of all genes clustered based on a dissimilarity measure. **(C)** Module stability test.

The WGCNA analysis identified seven gene co-expression modules (Figures 2A,B). We performed a module stability test using the same data set and confirmed that the modules identified in Figure 2A are persevered (Figure 2C) (Langfelder et al., 2011; Li et al., 2015). We further focused on four modules that had the highest correlation coefficients: the brown, turquoise, yellow, and red modules (Figure 2A). The full list of genes for each of the four modules is provided in Supplementary Table S2.

### 2.1 Differential Expression Analysis for FRDA Identifies Multiple NEM Genes to Be Dysregulated

We further investigated the transcriptional changes of genes included in the four modules in the eight NDDs to ensure that any differentially expressed gene (DEG) obtained is highly correlated to FRDA.

First, DEA of the total transcripts of an FRDA microarray data set (GSE11204) was performed. All DEGs were obtained regardless of whether they are NEM genes or not. Then, NEM genes that were identified as DEGs after analyzing the total transcripts were selected to focus on. The data set included samples from adults and children. We included each in the analysis separately and compared the outcome (Supplementary Table S3). We could identify 124 NEM DEGs in the adult FRDA data set (FRDA-adults) and 33 in the children’s data set (FRDA-children). The advantage of performing WGCNA with one data set and the DEA with another supported the outcome of the analysis in two different ways.

### 2.2 DEA for the Seven NDDs Identifies Common Genes With FRDA

We next performed DEA to identify novel NEM DEGs for AD (GSE153873), HD (GSE64810), PD (GSE68719), ALS (GSE124439), MS (GSE135511), CJD (GSE124571), and FTD (GSE153960). The most common pathological form of FTD (constituting about 50% of FTD cases) is frontotemporal lobar degeneration-TDP (FTLD-TDP), which is characterized by neuronal and glial TDP-43 inclusions (Ling et al., 2013). Therefore, we focused the analysis on FTLD-TDP. For the CJD data, we analyzed sporadic CJD samples (sCJD).

**FIGURE 3:**
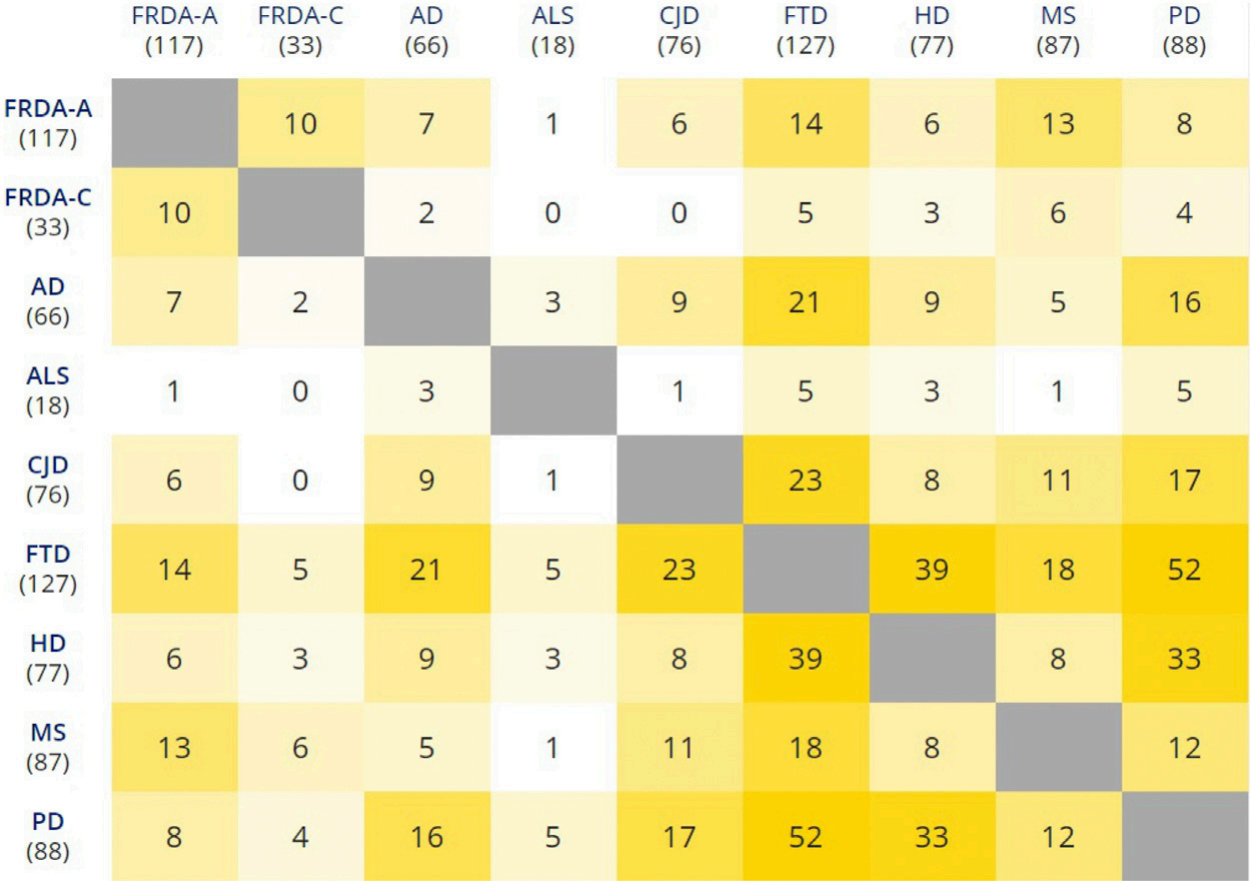
Pairwise intersections of the NEM DEGs belonging to the eight NDDs. FRDA-A, FRDA-adults; FRDA-C, FRDA-children. The matrix was plotted using the multiple list comparator of molbiotools (https://www.molbiotools.com).

In our analysis, we utilized a *p*-value of < .05 and the default log2 fold change (lfc = 0). Afterward, we selected only genes that were statistically significant in terms of adjusted *p*-value and if its |log2 fold change| > 0.6. Figure 3 represents the number of intersecting DEGs between all diseases. The full lists of differentially expressed NEM genes for the seven diseases are provided in Supplementary Tables S3–S10. These genes were selected after performing the analysis using the total transcripts. Slight variations in the number of DEGs between Figure 3 and the tables exist as Figure 3 does not include transcript variants.

We pinpointed the DEGs that were identified in one of the four selected modules and were also dysregulated in FRDA and at least one of the seven NDDs (Table 2). These DEGs were filtered again according to the adjusted *p*-value ≤ .03 to narrow down variations and increase the confidence in the hits. All the genes also code for proteins that have at least a mitochondrial localization confidence score 3/5 (Binder et al., 2014). Our analysis showed that ADH5, SLC25A36, BFSP-1, CASP1, HSPA1A, PYCARD, SHOX2, LDHB, UQCRB, DNAJC19, MRPL1, MTHFD2, NDUFA5, SCP2, TYMS, MAPK9, and PARG were dysregulated in either FRDA-children or FRDA-adults or both and additionally dysregulated in at least one of the seven NDDs (Table 2). In addition to the genes that were dysregulated in FRDA and other diseases, we found several genes to be specifically dysregulated in FRDA with a log2 fold change (lfc > 1). MAP2K1, PRDX3, NUBPL, PDE2A, KRAS, CHDH, AGR2, MTUS1, FSIP2, HOXB9, FSIP2, CRY2, and STARD13 were differentially expressed exclusively in FRDA-adults, and CREB1 was downregulated in FRDA-children only. Finally, ATF2 was downregulated in both FRDA-adults and FRDA-children.

**TABLE 2:** List of DEGs identified as common between FRDA and other NDDs. The genes included in this table were all identified in one of the four modules; turquoise, brown, red, and yellow. Upregulated (Up); lfc >0.6, downregulated (Down); lfc < −0.6. The confidence for the mitochondrial localization was determined *via* COMPARTMENTS (Binder et al., 2014). M, module; C, confidence.

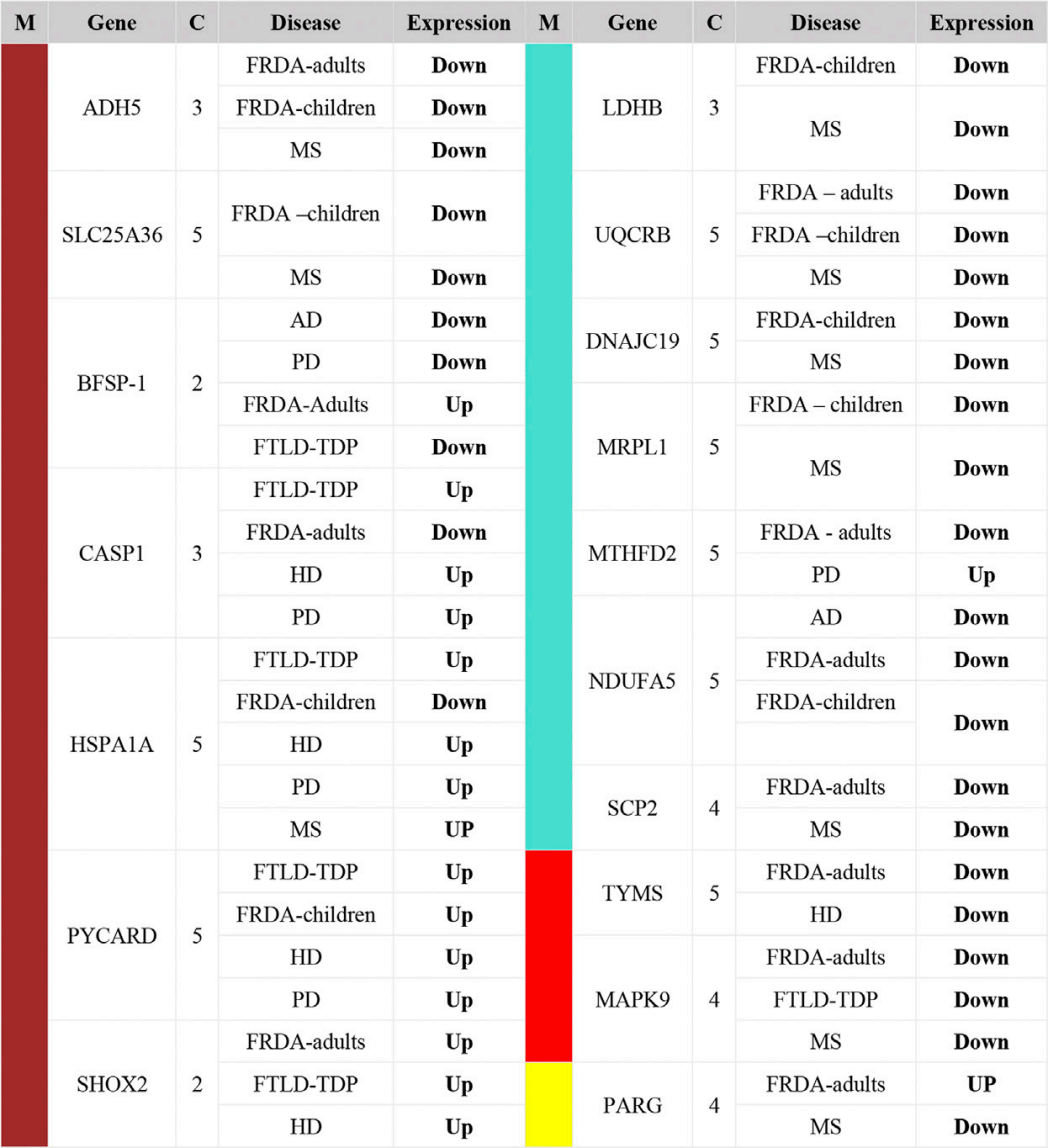

### 2.3 Pathway Enrichment Analysis of FRDA Selected Modules Reveals 13 Pathways

Associating particular genes with NDDs gives a lot of insight into the mechanisms behind pathogenesis. However, checking the enriched pathways in which the DEGs are involved provides a bigger picture. In different patients, specific genes are dysregulated, leading eventually to pathway impairment (Mohamed et al., 2021). Thus, investigating the enriched pathways ensures that different genes of the same pathway are taken into consideration.

**FIGURE 4:**
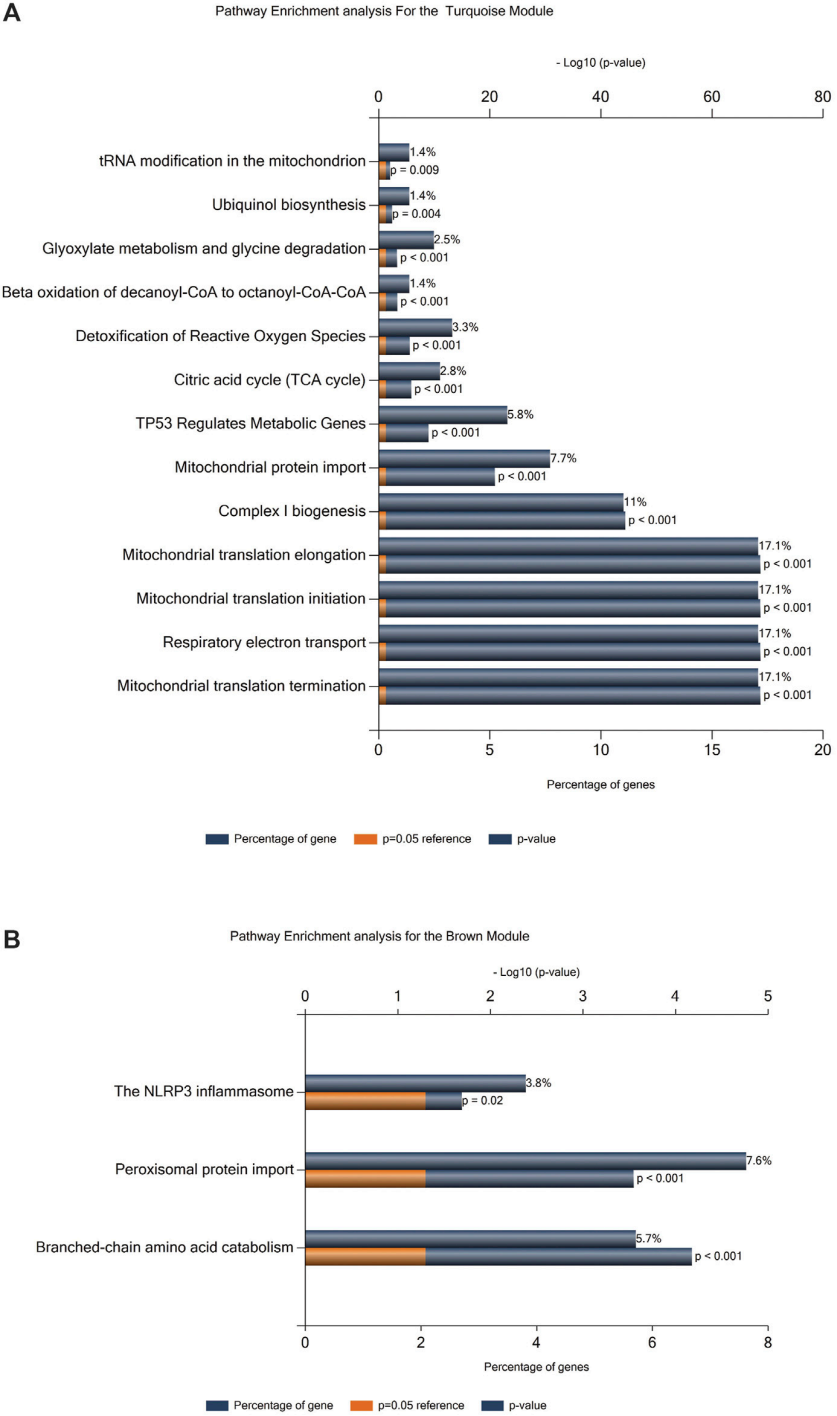
Pathway enrichment analysis. **(A)** Enrichment analysis for the turquoise module. **(B)** Enrichment analysis for the brown module.

To identify the pathways that are associated with FRDA samples, we employed pathway enrichment analysis. We selected the genes included in the turquoise and brown modules for the enrichment analysis as they were among the modules of a high correlation coefficient and also included a number of genes that grabbed our interest (Supplementary Table S2). The analysis was performed *via* FunRich^10^ software (version 3.1.1). Thirteen pathways were identified as enriched in the FRDA turquoise (Figure 4A) and three in brown modules (Figure 4B).

**FIGURE 5:**
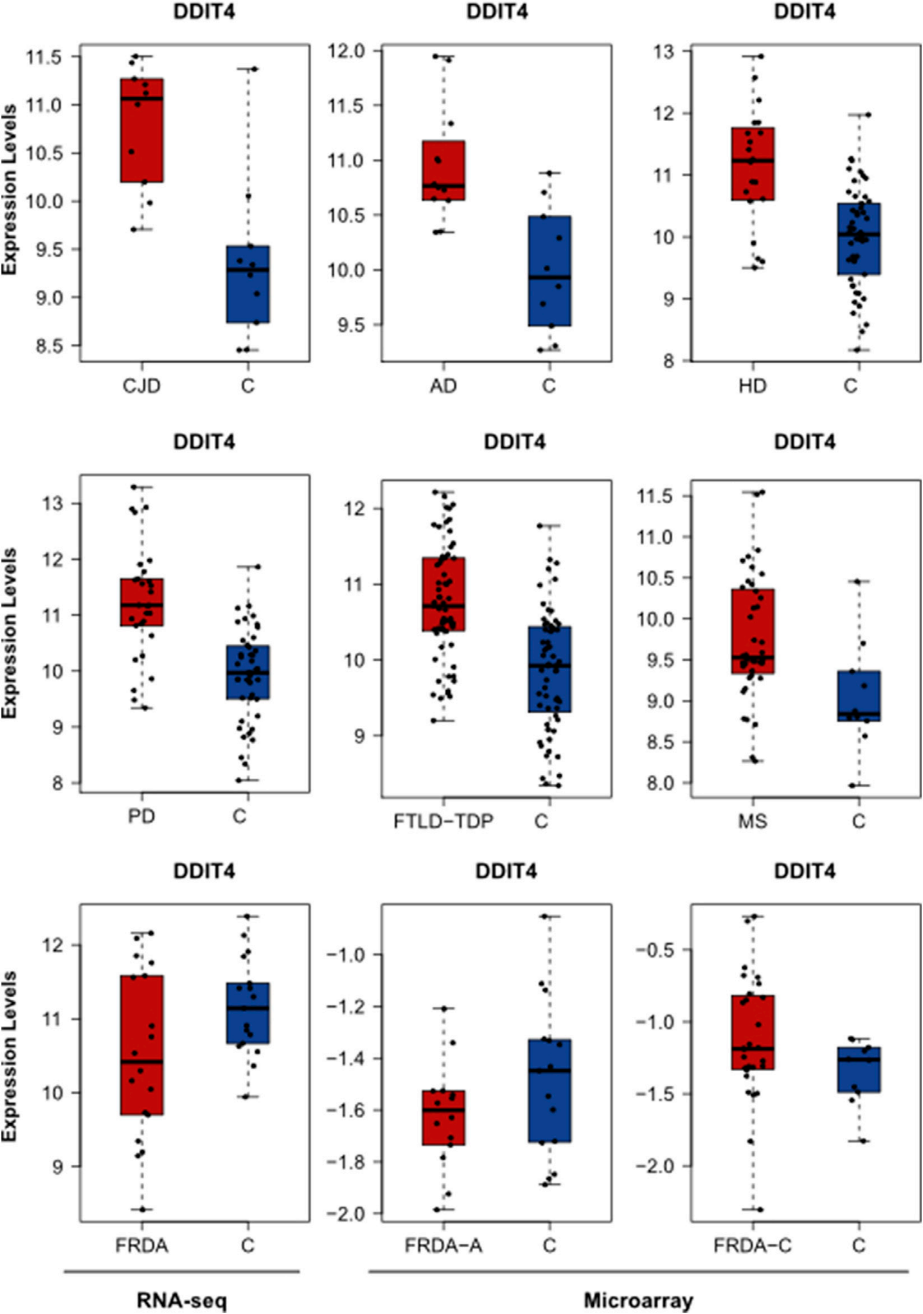
The expression levels of DDIT4 in AD, HD, PD, FTLD-TDP, MS, CJD, and FRDA. The expression levels of DDIT4 in FRDA samples of the RNA-seq data set are represented in addition to its expression in the FRDA-adults (FRDA-A) and FRDA-children (FRDA-C) data sets. C, ctrl.

We further investigated if our DEGs were involved in the enriched pathways. In Table 2, we focus on the DEGs that were common between FRDA and any of the other diseases. However, we realized that many important genes were not identified as DEGs in FRDA. Therefore, in order not to miss out on important genes that could be potentially linked to neurodegeneration, we list in Table 3 DEGs that were identified in any of the diseases and were also part of the enriched pathways and selected modules. The full list of genes involved in these pathways were obtained by the ReactomeContentService4R^11^ (version 1.0.0) package (Supplementary Table S11). The Venn tool^12^ in the Bioinformatics and Evolutionary Genomics web tools was also used to identify the common genes between pathways, modules, and DEGs. Twenty-six DEGs were identified in at least one of the NDDs and were also part of one of the modules and played a role in at least one of the enriched pathways (Table 3). The most interesting gene identified was the DNA damage inducible transcript 4 “DDIT4” as it was upregulated in six out of the eight diseases; AD, CJD, FTLD-TDP, HD, MS, and PD (Figure 5; Table 3). However, the gene was not differentially expressed in FRDA patients of the RNA-seq data set and also in FRDA-A and FRDA-C (Figure 5). The consistency of the data obtained from both data sets gives confidence that the dysregulation of this gene is not likely associated with FRDA.

**TABLE 3:** List of DEGs that are part of the enriched pathways of the brown and turquoise modules. Upregulated (Up); lfc > 0.6, downregulated (Down); lfc < −0.6. The confidence for the mitochondrial localization was determined via COMPARTMENTS (Binder et al., 2014). Genes represented in this table show adjusted *p*-value ≤ 0.03. M, module; C, confidence.

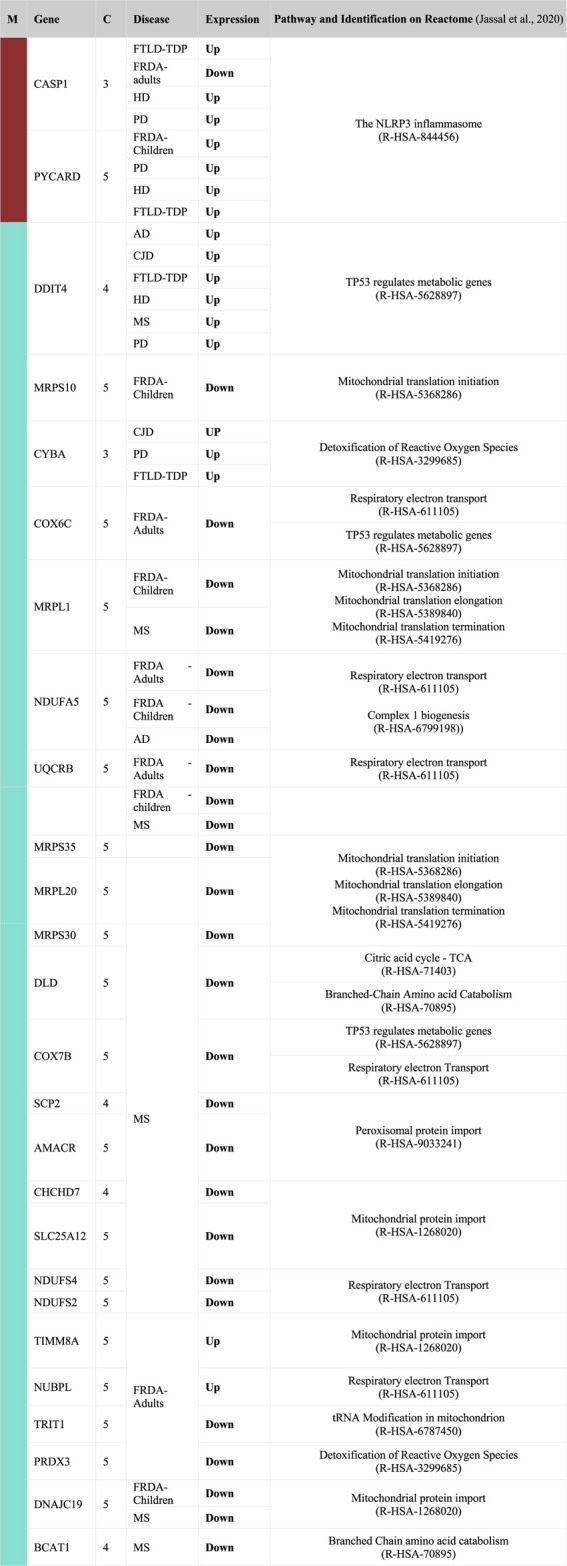

### 2.4 Six DEGs That Are Part of the Enriched Pathways and the Selected Modules Are Common Between FRDA and at Least One NDD

**FIGURE 6:**
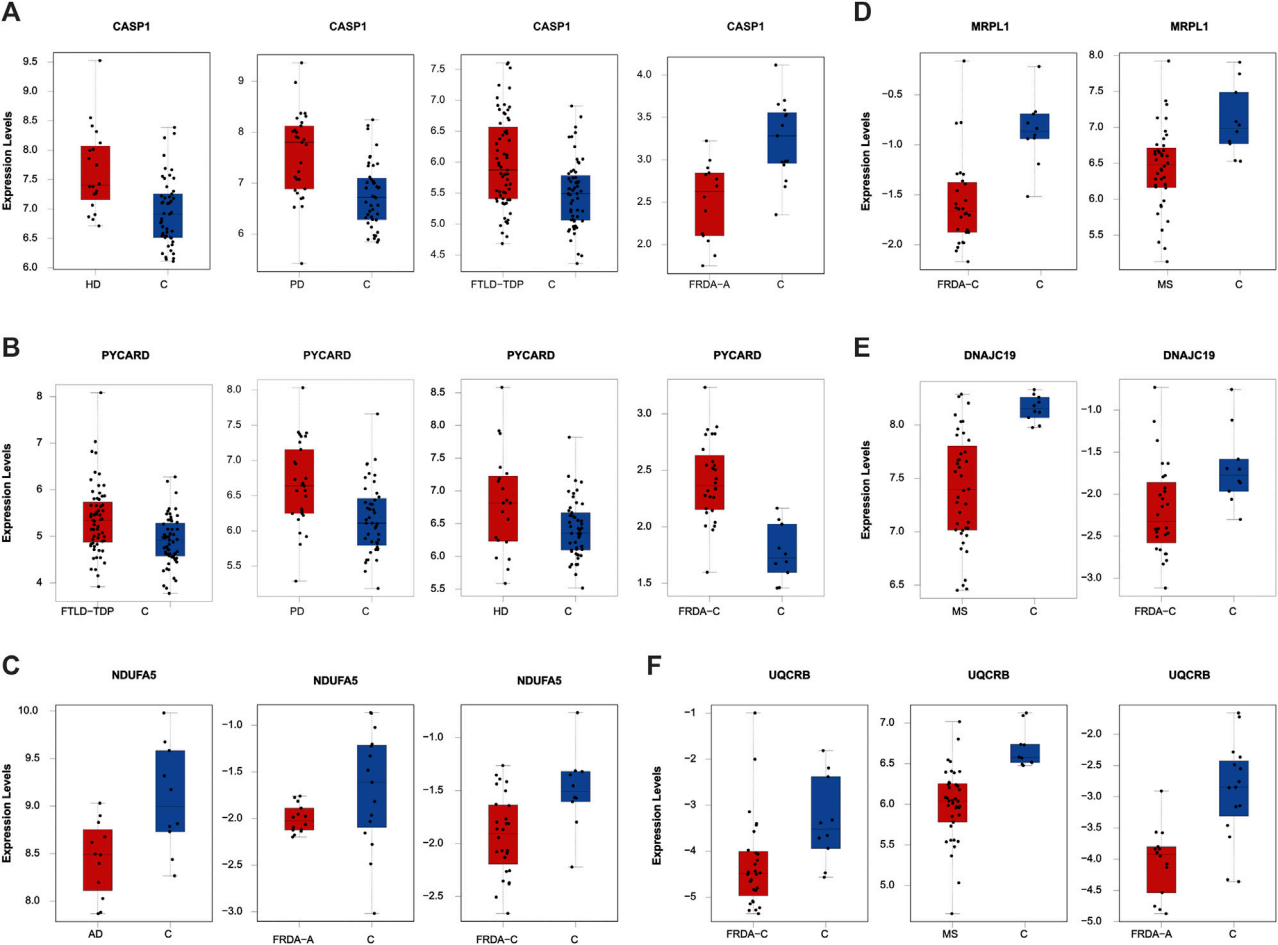
The expression levels of the DEGs common between FRDA and at least one NDD and additionally are part of the enriched pathways and the modules. **(A)** CASP1 in HD, PD, FLTD-TDP, and FRDA-adults. **(B)** PYCARD in FTLD-TDP, PD, HD, and FRDA-children. **(C)** NDUFA5 in AD, FRDA-adults, and FRDA-children. **(D)** MRPL1 in FRDA-children and MS. **(E)** DNAJC19 in MS and FRDA-children. **(F)** UQCRB in FRDA-children, MS, and FRDA-adults. FRDA-a, adult and FRDA-c, children and C, ctrl.

To finally identify the most relevant candidate genes that can potentially be used as drug targets in FRDA and other NDDs, we narrowed down all the DEGs to focus only on those which were part of the enriched pathways and the modules. Six genes were obtained; CASP1, PYCARD, DNAJC19, MRPL1, NDUFA5, and UQCRB (Table 4). Interestingly, PYCARD and CASP1 that function in the NLRP3 inflammasome pathway were upregulated in FTLD-TDP, HD, and PD. PYCARD was additionally upregulated in FRDA-children, and CASP1 was downregulated in FRDA-adults (Figures 6A,B). DNAJC19, MRPL1, NDUFA5, and UQCRB were all downregulated in FRDA and at least another NDD (Figures 6C–F; Table 4).

**TABLE 4:** List of DEGs common between FRDA and at least one NDD. The genes were also part of the enriched pathways and the modules. Upregulated (Up); lfc > 0.6, downregulated (Down); lfc < -0.6. The confidence for the mitochondrial localization was determined via COMPARTMENTS (Binder et al., 2014). Genes represented in this table show adjusted *p*-value ≤ 0.03. M, module; C, confidence.

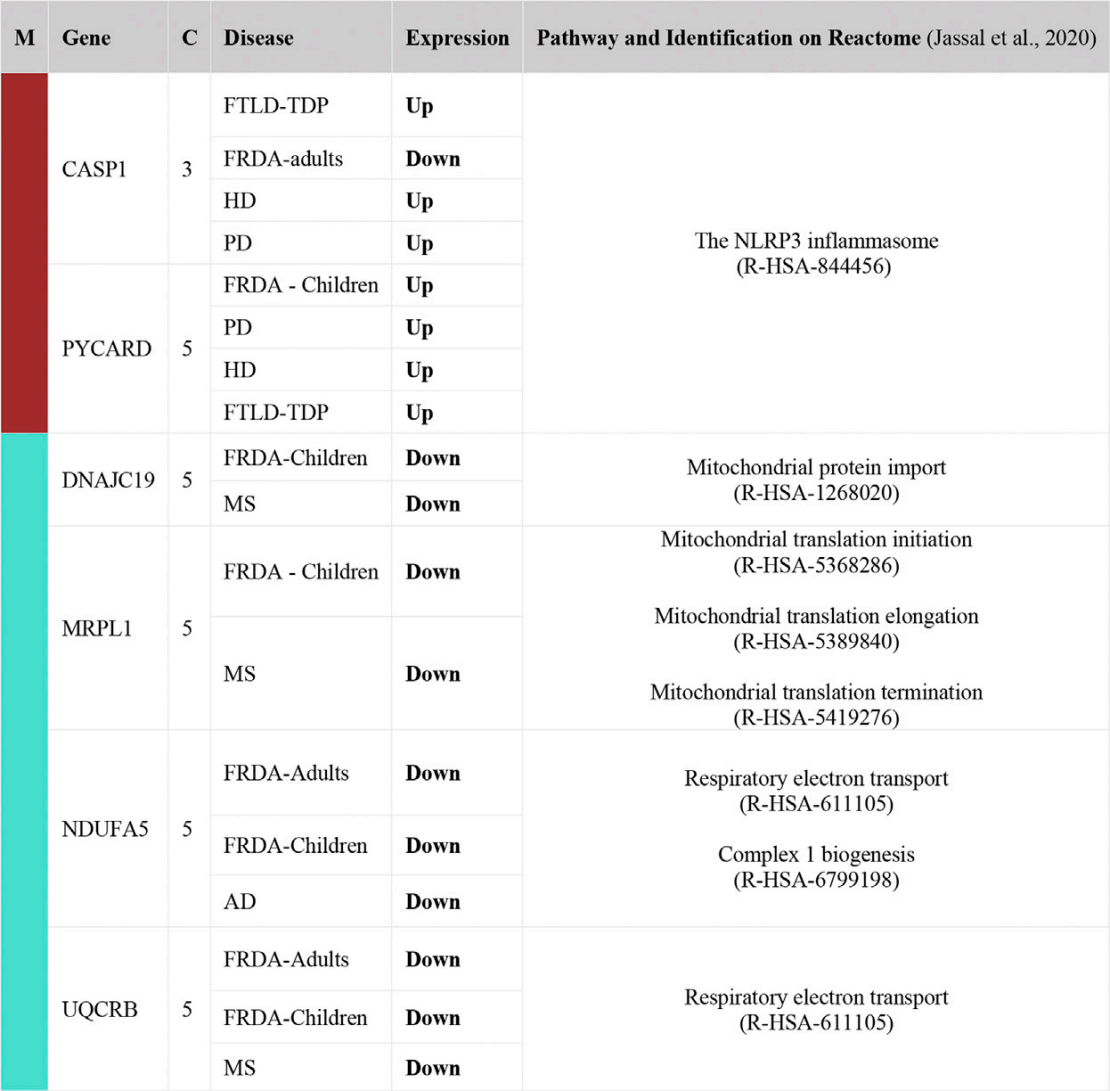

## 3 Discussion

Mitochondria are pivotal in ROS regulation, apoptosis, energy generation, and metabolic pathways, including oxidation of fatty acids, steroid, and heme biosynthesis. Consequently, it is not surprising that mitochondrial function is compromised in many diseases, particularly those associated with high-energy-demand organs, such as brain, heart, and skeletal muscles (Sangar et al., 2012; Watson et al., 2020). Our main interest was to identify NEM genes that are dysregulated in FRDA, which is still incurable and is challenged by the lack of treatment that can at least delay neurodegeneration despite numerous research efforts (Ocana-Santero et al., 2021). Here, we compare the transcriptomic changes between healthy controls and eight NDDs, including FRDA. We then selected the NEM genes to focus the analysis on them. We additionally narrowed down the selection to include genes coding for proteins that are imported to the mitochondria with high confidence (≥3) (Binder et al., 2014). Finally, we checked the pathways enriched in FRDA and investigated their enrichment in the seven diseases. Besides focusing on the transcription levels of specific genes, employing a pathway-centric approach enabled us to tackle different players that contribute to the robust pathway function.

Our data show transcriptional changes in PYCARD and CASP1 that function in the NLPR3 inflammasome pathway in several NDDs. CASP1 codes for caspase-1 that is involved in the activation of interleukin 1β (IL-1β), a key inflammatory mediator (Denes et al., 2012). PYCARD is a multiprotein complex that induces pyroptosis and neuroinflammation (Broz and Dixit, 2016; Lang et al., 2018). Caspase-1 usually becomes proteolytically active only after its dimerization with inflammasomes, such as PYCARD (Codolo et al., 2013). We show the overexpression of PYCARD in FRDA-children, FTLD-TDP, HD, and PD. On the other hand, FTLD-TDP, PD, and HD showed significant upregulation in CASP1, unlike FRDA-adults, as it was downregulated. Our results align with recent studies that show that NLRP3 inflammasomes can trigger neuroinflammation through a wide range of stimuli (Yang et al., 2019). Inflammasomes are also activated by oxidative stress and excessively activated microglia, both of which contribute to the pathogenesis and progression of FTLD-TDP, HD, and PD (Lefkowitz and Lefkowitz, 2008; Hernandez et al., 2017; Carmo et al., 2018; Voet et al., 2019). Furthermore, studies performed on HD brain samples detected an elevation and excessive activation of caspase-1, and its inhibition was shown to slow down disease progression (Caron et al., 2018). This is also reported for other NDDs (Yang et al., 2019). Even though CASP1 was not overexpressed in the FRDA data set we analyzed, a previous study investigating frataxin-deficient lymphoblasts from FRDA patients shows its upregulation (Tan et al., 2003). However, we think we did not detect an overexpression in CASP1 in FRDA as its overexpression is usually associated with diseases linked to toxic aggregation accumulating in FTD, HD, and PD patients’ cells, which are not reported for FRDA (Wellington et al., 1998).

We interestingly found transcriptional changes in several genes involved in mitochondrial protein synthesis pathways, including translation initiation, elongation, and termination (Mai et al., 2017; Kummer and Ban, 2021). The small 28S and large 39S ribosomal subunits are coded for by the human mitochondrial ribosomal protein (MRP) gene family (Gopisetty and Thangarajan, 2016). Changes in the expression of MRPs destroy ribosomal composition, disrupt mitochondrial metabolic functions, and result in a multitude of mitochondrial diseases (Huang et al., 2020). MRP genes are also associated with neuronal differentiation and development (Gopisetty and Thangarajan, 2016). For example, MRPL2, MRPL14, MRPS10, MRPS18A, and MRPS18B are candidate genes for spinocerebellar ataxia with blindness and deafness (Papapetropoulos et al., 2006). Our results show that many of the MRPs are differentially expressed in MS and FRDA. In MS, MRPS35, MRPS30, MRPL1, and MRPL15 were downregulated, and MRPS28, MRPS35, and MRPL19 were downregulated in FRDA-adults. Interestingly, MRPL52 was upregulated in FRDA-adults. In FRDA-children, MRPL1 and MRPS10 were also downregulated. In agreement with this finding, MRPL family members were previously reported to be significantly underexpressed in FRDA fibroblasts (Napierala et al., 2017).

Changes in expression levels of genes functioning in mitochondrial protein import were also observed in several diseases. SLC25A12 and DNAJC19 were downregulated in MS. The latter was also downregulated in FRDA-children. TIMM8A on the contrary was upregulated in FRDA-adults in our analysis. Finally, CHCHD7 was also downregulated in MS. Mutations in DNAJC19 are previously associated with dilated cardiomyopathy in ataxia syndrome (Davey et al., 2006). Defects in TIMM8A are also linked to the deafness dystonia NDD (Koehler et al., 1999; Hoogenraad et al., 2002).

The enrichment analysis revealed several genes involved in the citric acid (TCA) cycle, respiratory electron transport, and Complex I Biogenesis to be differentially expressed in several NDDs. Alterations in glucose metabolism can affect the maintenance of neurotransmission and neuronal function and impact the ability to learn and memorize (Yan et al., 2020). It is also reported that the mitochondrial citric acid cycle can regulate the pathogenesis of neuroinflammation and neurodegeneration (Garabadu et al., 2019). The identified genes that play a role in one or more of these pathways included COX6C, UQCRB, and NDUFA5. COX6C was downregulated in FRDA-adults, and UQCRB was downregulated in FRDA-adults, FRDA-children, and MS. NDUFA5 was also downregulated in FRDA-adults, FRDA-children, and AD. NUBPL was, on the other hand, upregulated in FRDA-adults. Furthermore, NDUFA5 was reported to be differentially expressed in FRDA patients (Télot et al., 2018). UQCRB was also previously reported to be downregulated in FRDA (Télot et al., 2018). We did not detect changes in COX6C in AD; however, downregulation of the gene was previously linked to AD (Bi et al., 2018).

Among the pathways identified to be enriched in FRDA is “TP53 regulates metabolic genes.” Many recent studies show that p53 plays a role in neuropathogenesis and have found that increased levels of p53 are linked to neuronal cell death (Eun et al., 2010; Proctor and Gray, 2010). The DNA damage inducible transcript 4 “DDIT4” protein, also known as REDD1, regulates mTOR signaling (Cam et al., 2014). The DDIT4 gene was found to be upregulated in AD, CJZ, FTLD, HD, MS, and PD. DDIT4 was the highest elevated transcript (98-fold), and its encoded protein, RTP801, was considerably increased in cellular models of PD (Ryu et al., 2005). DDIT4 is also associated with AD and HD (Kim et al., 2003; Martín-Flores et al., 2020; Pérez-Sisqués et al., 2021).

Other DEGs playing a role in enriched pathways were identified. For example, SCP2 and AMACR functioning in peroxisomal protein import were downregulated in MS. CYBA functioning in detoxification of ROS was upregulated in HD, PD, and CJD. TRIT1, which modifies both cytosolic and mitochondrial tRNAs (Suzuki et al., 2011; Khalique et al., 2020), was also downregulated in FRDA-adults.

HSPA1A, which codes for heat shock protein 70 (HSP70), was upregulated in MS, FTD, HD, and PD. On the contrary, it was downregulated in FRDA-children. Expression levels of HSPs can change according to the response and function of the heat shock protein, the disease, the affected cell, and even the brain region (Leak, 2014). It is speculated that the rise in the expression of HSP70 in neurodegenerative conditions is to help slow the disease progression and delay aging as shown in PD, HD, and FTD (Lazarev et al., 2013; Calderwood and Murshid, 2017). In FRDA, on the other hand, the reason for the significant decline of HSP70 expression is because HSP70 is one of the essential components that facilitates the mitochondrial FeS cluster assembly, which is disrupted as a result of frataxin mutation (Stemmler et al., 2010). Finally, BCAT-1 was downregulated in MS. Previous reports show that knockdown of BCAT-1 demonstrate PD-like features, including progressive motor deficits and neurodegeneration developing with age (Mor et al., 2020).

ALS surprisingly showed very few similarities in the transcriptome profile of NEM genes when compared with the other NDDs. The total NEM DEGs in ALS were relatively fewer than the average of the other diseases. However, it still shares one NEM DEG with FRDA-adults, CJD, and MS, three with each of AD and HD as well as five NEM DEGs with each of FTD and PD. However, the genes were not part of the turquoise or brown modules and, therefore, were not focused on in the analysis. The full list of ALS DEGs is available in Supplementary Table S8.

**FIGURE 7:**
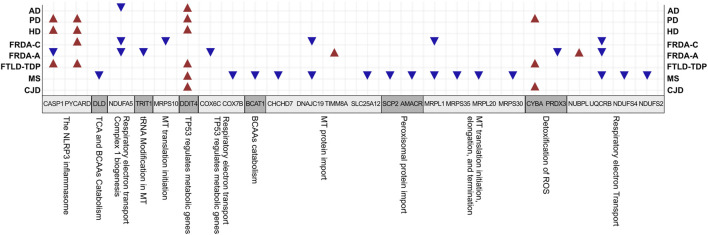
A summary for the interesting genes and associated pathways identified in all diseases. BCAAs, branched-chain amino acids; TCA, tricarboxylic acid cycle; MT, mitochondrial.

The findings support an association of transcriptional changes in NEM genes with NDDs (Figure 7). The identified DEGs could possibly act as novel biomarkers, and the proteins for which they code can act as drug targets for the NDDs we discussed. Indeed, regulation of several players is already reported. For example, capase-1 is shown to be inhibited by VX-765 (Belnacasan). Belnacasan was able to stop the accumulation of amyloid β protein, prevent axonal neuronal degeneration, reduce release of inflammasome-associated cytokines, and ameliorate memory and cognitive function when tested on AD mice model (Flores et al., 2018). The same drug also inhibited proinflammatory caspase activation in MS mice models and reduced neurodegeneration (McKenzie et al., 2018). NDUFA5 is also shown to be upregulated by Pioglitazone (Coletta et al., 2009). Moreover, synthetic small molecules that specifically target UQCRB were also designed and showed an anti-angiogenic effect, causing repression of tumor growth in mouse xenograft models (Jung et al., 2014). Recent data also show that dexamethasone induces a novel epigenetic function for HDAC4, which involves switching on DDIT4 expression in ataxia telangiectasia and, consequently, increasing the protein levels (Ricci et al., 2020). Overall, our findings open doors for investigating the drugability of the players and further testing of known inhibitors on the NDDs discussed.

## 4 Conclusion

Despite the complex nature of NDDs, continuous research efforts are directed toward finding a cure to halt the escalation of symptoms and provide a definitive treatment. In this study, we investigate the transcriptomic changes in NEM genes in FRDA and seven other NDDs. Our results provide an expanded overview showing association of transcriptional dysregulation in NEM genes with all NDDs analyzed. In addition, pathways crucial for proper mitochondrial function were also associated with the DEGs. The NEM genes pinpointed in this study can serve as potential markers and drug targets, which can be further investigated in animal models and also in individual patients to confirm their dysregulation in a personalized manner.

## 5 Methods

### 5.1 Obtaining the Full List of NEM Genes

The full Ensembl IDs list of NEM genes was retrieved from (Eslamieh et al., 2017). The authors utilized the BioMart^13^tool to mine the ENSEMBL genome database^14^ (Ensembl) and select the nuclear encoded genes with mitochondrial annotation; specified by the Gene Ontology ID GO: 0005739 (mitochondrion). We obtained the confidence score for evidence of mitochondrial localization for each gene from COMPARTMENTS subcellular localization data section in GeneCards^15^ (Binder et al., 2014). Afterward, all the Ensembl gene IDs were converted to gene symbols using the org.Hs.eg.db R package^16^ (version 3.12.0).

### 5.2 Identification of Gene Co-Expression Modules Using WGCNA

Normalized counts for the FRDA RNA-seq data set were downloaded from GEO^17^ under the accession number GSE104288. The data set included 35 samples from primary fibroblast cell lines derived from 18 FRDA patients and 17 control individuals.

Co-expression networks facilitate methods to construct a network-based gene screening, which can be utilized to pinpoint candidate biomarkers and therapeutic targets. In this study, the WGCNA^18,^ ^19^ package (Langfelder and Horvath, 2008) was used to construct gene co-expression networks for the expression profiles of FRDA patient and control samples. The modules of highly correlated genes were explored among samples for relating modules to external sample traits. The pick soft threshold function was used to build a scale-free network, and soft power *β* = 6 was selected. Subsequently, the weighted adjacency matrix was constructed, followed by transforming adjacencies and correlations into a topological overlap matrix (TOM). Then, dissimilarity (1-TOM) was calculated. The genes hierarchical clustering analysis was then done using 1-TOM as the distance measure (Yip and Horvath, 2007). A dynamic tree cut algorithm was used to detect modules with a minimum module size of 30 and a minimum cut height of 0.75. Afterward, for identifying functional modules in the co-expression network, the associations between modules and module-traits were calculated according to a previous study (Yip and Horvath, 2007). Ultimately, the highly correlated coefficient modules were recognized as candidates relevant to module traits, and two of them were selected for subsequent analyses. Besides this, the WGCNA function (module preservation) was used to test the module preservation; GSE104288 was randomly divided into training and test sets using the sample function in package base (version 4.1.1). Two parameters for module preservation statistics were defined as follows: Zsummary, the average of Z-scores calculated for connectivity and density measures and the statistic median rank, and the average calculation of median ranks for connectivity and density measures of each module. Modules with a Z-score ≥ 10 are considered as highly preserved modules, and 2 < Z-score < 10 indicate good stability (Langfelder et al., 2011; Li et al., 2015).

### 5.3 DEA of Microarray Data

All data sets were obtained from the National Center for Biotechnology Information (NCBI) Gene Expression Omnibus (GEO). The microarray data analysis was done using limma package (version 3.46.0) (Ritchie et al., 2015) to perform the default analysis workflow on each data set individually. The moderated t-statistic is used for significance analysis. The test works as the ordinary *t*-test with an exception that the standard errors are moderated across genes, i.e., embraced to a mutual value, utilizing a simple Bayesian model (Ritchie et al., 2015). The result was filtered based on adjusted *p*-value < .05 and |log2 fold change| > 0.6. The full list of DEGs identified before selecting the NEM genes are indicated in Supplementary Table S12.

#### 5.3.1 FRDA

Raw FRDA expression data (GSE11204) was obtained. The expression data for peripheral blood samples includes 14 FRDA adults and 15 healthy adults (mean age 36.8 and 25.4, respectively), and the remaining samples belong to 28 FRDA-children and 10 healthy controls with the mean age of 13.5 and 20.3, respectively. Then background correction with an offset = 10 for adult data and = 16 for children’s data was performed.

#### 5.3.2 MS

We retrieved the MS data set (GSE135511) of the motor cortex from 20 postmortem MS brains with and without substantial meningeal inflammation as well as 10 non-neurological controls. Both MS disease groups were analyzed together as one inclusive MS group versus the control group.

#### 5.3.3 CJD

The normalized gene expression CJD data (GSE124571) were obtained. Samples were collected from the frontal cortex (middle frontal gyrus) of 10 sCJD brains (five each from the two commonest disease subtypes; MM1 and VV2) and 10 controls without any history of neurological or psychiatric illnesses. The data was log2 transformed prior to the DEA.

### 5.4 DEA of RNA-Seq Data

All data sets were downloaded from GEO. The RNA-Seq DEA was done using DESeq2 package (version 1.30.0) to perform the default analysis workflow on each data set separately (Love et al., 2014). The DESeq2 package evaluates the variance-mean dependence in the count data to then determine the DEGs based on a negative binomial distribution. The differential analysis significance testing is based on a two-tailed Wald test. The main approach for extracting the DEGs in all the following data sets is primarily through setting the DESeq2 result function with a threshold (Benjamini-Hochberg adjusted *p*-value < .05). Afterward, the produced results were filtered based on adjusted *p*-value < .05 and |log2FC| > 0.6. Then, the non-NEM DEGs were removed from the filtered results. In case the obtained data set consisted of Ensembl IDs, they were converted into gene symbols using org.Hs.eg.db R package (version 3.12.0). The full list of DEGs identified before selecting the NEM genes are indicated in Supplementary Table S12.

#### 5.4.1 AD

The AD data set (GSE153873) was downloaded. We only used the AD group (12 samples) and old controls (10 samples). The age range of both groups was 61–79 years. Moreover, the postmortem brain tissue samples were obtained from the lateral temporal lobe (Brodmann area 21 or 20) (Nativio et al., 2020). This RNA-seq data set consisted of the expression raw counts of 27,133 gene symbols.

#### 5.4.2 HD

The HD data set (GSE64810) contained an HD group (20 samples) and a healthy controls group (49 samples). The age of healthy controls was 36–106 years, and the HD patients’ aged around 40–75 years (Labadorf et al., 2015). The RNA-seq data was obtained from postmortem human prefrontal cortex samples from Brodmann Area 9 (BA9) (Labadorf et al., 2015). The obtained data set consisted of the expression raw counts for 27,282 transcripts provided in Ensembl gene ID.

#### 5.4.3 PD

The PD data set (GSE68719) consisted of 29 patient samples and 44 healthy controls. The age of the healthy controls ranged from 46 to 97 years, and the PD patients’ age ranged from 64 to 95 years (Dumitriu et al., 2016). The brain tissue samples were obtained from postmortem human brain samples from the prefrontal cortex Brodmann Area 9 (BA9) (Dumitriu et al., 2016). The provided RNA-seq data set contained expression raw counts for 27,282 transcripts provided as Ensembl gene ID.

#### 5.4.4 ALS

The ALS RNA-seq data set (GSE124439) was downloaded in the form of multiple text files, one for each patient’s gene counts. All the data set transcriptomes are obtained from postmortem cortex samples by the NYGC ALS Consortium (Tam et al., 2019). The selected samples in this data set consisted of a total of 165 samples (i.e., ALS spectrum patients, 148 samples, and non-neurological controls, 17 samples) were selected for the analysis. The gene expression count matrix consisted of 27,961 gene symbols.

#### 5.4.5 FTD/FTLD-TDP

The FTD RNA-seq data set (GSE153960) was retrieved in the form of one text file, including the raw gene counts matrix. The data was filtered to only select for FTLD-TDP (i.e., frontotemporal lobar degeneration with TDP-43 inclusions) patients and healthy controls. The RNA-seq library was extracted from postmortem brain tissue samples by the NYGC ALS Consortium (Prudencio et al., 2020). To reduce the intersample variability, only frontal and temporal cortices were selected from the tissue types and NovaSeq from the sequencing platform. After filtration, the number of samples collectively consisted of 122 samples (59 control and 63 FTLD-TDP patients). The data set contained 58,929 transcript expression counts.

### 5.5 Pairwise Intersection Matrix

The pairwise intersection matrix of the NEM DEGs in the eight NDDs was plotted using the multiple list comparator of molbiotools.

### 5.6 Pathway Enrichment Analysis for Modules of Interest

FunRich tool^1^ (version 3.1.1 March 2017) was used to explore the enriched pathways in the brown and turquoise modules with a cutoff criterion of adjusted *p*-value < .05 (Pathan et al., 2015). Afterward, the ReactomeContentService4R (version 1.0.0) was used to identify the genes included in the aforementioned enriched pathways based on the Reactome database (Jassal et al., 2020). Afterward, the Venn tool^2^ in the Bioinformatics and Evolutionary Genomics web tools was also used to identify the overlapping genes between pathways, modules, and disease DEGs.

## Data Availability

The datasets presented in this study can be found in online repositories. The names of the repository/repositories and accession number(s) can be found in Table 1.

## Footnotes

10
http://www.funrich.org/

11
https://bioconductor.org/packages/release/bioc/html/ReactomeContentService4R.html

12
http://bioinformatics.psb.ugent.be/webtools/Venn/

13
http://www.biomart.org/

14
https://useast.ensembl.org/index.html

15
https://www.genecards.org/

16
https://bioconductor.org/packages/release/data/annotation/html/org.Hs.eg.db.html

17
https://www.ncbi.nlm.nih.gov/geo/

18
https://cran.r-project.org/web/packages/WGCNA/index.html

19
https://www.molbiotools.com

## Supplementary Material

Supplementary Table S1 List of nuclear-encoded mitochondrial genes with their confidence values of being localized to the mitochondria. The confidence for the mitochondrial localization was determined *via* COMPARTMENTS (Binder et al., 2014).

Supplementary Table S2 List of co-expressed genes in the identified modules of NEM genes in FRDA patients.

Supplementary Table S3 NEM DEGs in FRDA patients. Adjusted *p*-value < .05 and |log2 fold change| > 0.6.

Supplementary Table S4 NEM DEGs in MS patients. Adjusted *p*-value < .05 and |log2 fold change| > 0.6.

Supplementary Table S5 NEM DEGs in HD patients. Adjusted *p*-value < .05 and |log2 fold change| > 0.6.

Supplementary Table S6 NEM DEGs in PD patients. Adjusted *p*-value < .05 and |log2 fold change| > 0.6.

Supplementary Table S7 NEM DEGs in AD patients. Adjusted *p*-value < .05 and |log2 fold change| > 0.6.

Supplementary Table S8 NEM DEGs in ALS patients. Adjusted *p*-value < .05 and |log2 fold change| > 0.6.

Supplementary Table S9 NEM DEGs in FTLD-TDP patients. Adjusted *p*-value < .05 and |log2 fold change| > 0.6.

Supplementary Table S10 NEM DEGs in CJD patients. Adjusted *p*-value < .05 and |log2 fold change| > 0.6.

Supplementary Table S11 List of genes involved in the identified pathways. The genes were retrieved from Reactome database (Jassal et al., 2020).

Supplementary Table S12 Total DEGs identified in all diseases before selecting NEM genes. Adjusted *p*-value < .05 and |log2 fold change| > 0.6.
